# Supplementary material for: Antioxidant and Antiapoptotic Effects of a Turraea fischeri Leaf Extract on Cryopreserved Goat Sperm
Source: Animals (Basel). 2021 Sep 29;11(10):2840. doi: 10.3390/ani11102840 (PMC8532954; doi:10.3390/ani11102840)
Supplement: Supplementary file 1 [file animals-11-02840-s001.zip › animals-1335614-supplementary.pdf]

Antioxidant and Antiapoptotic Effects of a *Turraea fischeri* Leaf Extract on Cryopreserved Goat Sperm

HPLC-MS/MS

The leaf extract was analysed using high performance liquid chromatography-mass spectrometry. The LC system was Thermofinigan (Thermo Electron Corporation, USA) coupled with an LCQ-Duo ion trap mass spectrometer with an ESI source (ThermoQuest). The separation was achieved using a C18 reversed-phase column (Zorbax Eclipse XDB-C18, rapid resolution, 4.6 × 150 mm, 3.5 µm, Agilent, USA). A gradient of water and acetonitrile (ACN) (0.1% formic acid each) was applied from 5% to 30% ACN in 60 min in flow rate of 1 ml/min with a 1:1 split before the ESI source. The MS was operated in the following conditions: capillary voltage (−10 V), the source temperature was set at 200 °C, and nitrogen was used as a sheath and auxiliary gas at a flow rate of 80 and 40 (arbitrary units), respectively in the negative mode. MS/MS fragmentation was recorded with collision energy of 35%. The ions were detected in a full scan mode and mass range of 50–2000 *m/z* and finally the machine was controlled using Xcalibur software (Xcalibur 2.0.7, Thermo Scientific) [1].

Total phenolic content (TPC)

The TPC of the extracts was quantified using the FolinCiocalteu method adapted to 96 well-plates. 20 µL of the extract, dissolved appropriately in distilled water, was mixed with 100 µL FC reagent (freshly diluted 1/10 with distilled water). After 5 min incubation at room temperature, 80 µL of 7.5% Na_2CO_3 solution was added. The incubation stands for 30 min at room temperature in the dark with slightly shaking. Then, the absorbance of the solution was measured at 735 nm in a microplate reader (Biochrom Asys UVM 340) against blank. Gallic acid was used as a standard polyphenol. The results (mean of triplicate analyses) are expressed as gallic acid equivalent (GAE) in mg/g of extract [2].

Antioxidant assays

DPPH radical-scavenging assay

DPPH assay was performed according to the method described by Clarke et al. [3], with slight modifications. Briefly, 200 µL of plant extract, diluted appropriately in methanol in a concentration range from 0.24 to 500 mg/mL, was mixed with 100 µL of 0.2 mM DPPH in methanol in wells of 96-well plates. The plates were kept in the dark for 30 min, thereafter the absorbance of the solution was measured at 515 nm in a Biochrom Asys UVM 340 Microplate Reader. Appropriate blanks, methanol and standards (ascorbic acid solutions in methanol) were analysed simultaneously. The scavenging activity (in %) was calculated using the following equation:

DPPH scavenging $100 \times \frac{[(\text{Abs sample} + \text{DPPH}) - (\text{Abs sample blank})]}{[(\text{Abs DPPH}) - (\text{Abs methanol})]}$ =

The IC_{50} value is defined as the amount of extract needed to scavenge 50% of DPPH radicals. All analyses were performed in triplicate [2].

Ferric reducing antioxidant power assay

The ferric reducing antioxidant power (FRAP) assay was carried out according to the previously reported procedure with minor modifications [4]. Each sample was dissolved in methanol to prepare the stock solution (1 mg/ml). Briefly, the working FRAP reagent was prepared freshly by mixing 300 mM acetate buffer (pH 3.6), a solution of 10 mM 2,4,6- tripyridyl-s-triazine (TPTZ) in 40 mM hydrochloric acid and 20 mM ferric chloride at 10 : 1 : 1 (v/v/v). 20 µL of each extract was mixed with 180 µL FRAP reagent in wells of 96- well plates. The mixture was then incubated for 6 min at 37 °C, and the absorbance was measured at 595 nm in a microplate reader (Biochrom Asys UVM 340). Appropriate blanks of plant extract and of FRAP reagent lacking TPTZ (to correct the colors of the extracts) were run, together with quercetin (in methanol), and ferrous sulphate heptahydrate ($FeSO_4.7H_2O$) was used as a standard. FRAP activity was calculated as ferrous equivalents (FE), the concentration of extract/quercetin which produced an absorbance value equal to that of 1 mM FeSO4 [2].

References

1. Hepatoprotective and Hypoglycemic Effects of a Tannin Rich Extract from Ximenia Americana Var. Caffra Root. *Phytomedicine* **2017**, *33*, 36–42, doi:10.1016/j.phymed.2017.07.003.
2. Ghareeb, M.A.; Mohamed, T.; Saad, A.M.; Refahy, L.A.-G.; Sobeh, M.; Wink, M. HPLC-DAD-ESI-MS/MS Analysis of Fruits from Firmiana Simplex (L.) and Evaluation of Their Antioxidant and Antigenotoxic Properties. *Journal of Pharmacy and Pharmacology* **2018**, *70*, 133–142.
3. Clarke, G.; Ting, K.N.; Wiart, C.; Fry, J. High Correlation of 2, 2-Diphenyl-1-Picrylhydrazyl (DPPH) Radical Scavenging, Ferric Reducing Activity Potential and Total Phenolics Content Indicates Redundancy in Use of All Three Assays to Screen for Antioxidant Activity of Extracts of Plants from the Malaysian Rainforest. *Antioxidants* **2013**, *2*, 1–10.
4. Qader, S.W.; Abdulla, M.A.; Chua, L.S.; Najim, N.; Zain, M.M.; Hamdan, S. Antioxidant, Total Phenolic Content and Cytotoxicity Evaluation of Selected Malaysian Plants. *Molecules* **2011**, *16*, 3433–3443, doi:10.3390/molecules16043433.
